# Metabolic signature of *Mycobacterium avium* subsp. *paratuberculosis* infected and infectious dairy cattle by integrating nuclear magnetic resonance analysis and blood indices

**DOI:** 10.3389/fvets.2023.1146626

**Published:** 2023-04-17

**Authors:** Andrea Massaro, Alessandra Tata, Ivana Pallante, Valentina Bertazzo, Massimo Bottazzari, Laura Paganini, Brunella Dall'Ava, Annalisa Stefani, Jeroen De Buck, Roberto Piro, Nicola Pozzato

**Affiliations:** ^1^Laboratorio di Chimica Sperimentale, Istituto Zooprofilattico Sperimentale delle Venezie, Vicenza, Italy; ^2^Laboratorio di Medicina Forense Veterinaria, Istituto Zooprofilattico Sperimentale delle Venezie, Vicenza, Italy; ^3^Medicina di Laboratorio, Istituto Zooprofilattico Sperimentale delle Venezie, Legnaro, Italy; ^4^Laboratorio di Diagnostica Clinica e Sierologia di Piano, Istituto Zooprofilattico Sperimentale delle Venezie, Verona, Italy; ^5^Faculty of Veterinary Medicine, University of Calgary, Calgary, AB, Canada

**Keywords:** paratuberculosis, LASSO, NMR, metabolomics, prediction model, biomarker, pathway analysis, *Mycobacterium avium* subsp. *paratuberculosis*

## Abstract

The early diagnosis of *Mycobacterium avium* subsp. *paratuberculosis* (MAP) is one of the current challenges of farmers and veterinarians. This work aimed to investigate the changes in metabolic levels associated with natural MAP infection in infected and infectious dairy cattle. The study included sera from 23 infectious/seropositive, 10 infected but non-infectious/seronegative, and 26 negative Holstein Fresian cattle. The samples were selected from a collection of samples gathered during a prospective study. The samples were analyzed by quantitative nuclear magnetic resonance (NMR) spectroscopy and routine blood chemistry. The blood indices and the ^1^H NMR data were concatenated by low-level data fusion, resulting in a unique global fingerprint. Afterwards, the merged dataset was statistically analyzed by the least absolute shrinkage and selection operator (LASSO), which is a shrinkage and selection method for supervised learning. Finally, pathways analysis was performed to get more insights on the possible dysregulated metabolic pathways. The LASSO model achieved, in a 10 time repeated 5-fold cross-validation, an overall accuracy of 91.5% with high values of sensitivity and specificity in classifying correctly the negative, infected, and infectious animals. The pathway analysis revealed MAP-infected cattle have increased tyrosine metabolism and enhanced phenylalanine, tyrosine and tryptophan biosynthesis. The enhanced synthesis and degradation of ketone bodies was observed both in infected and infectious cattle. In conclusion, fusing data from multiple sources has proved to be useful in exploring the altered metabolic pathways in MAP infection and potentially diagnosing negative animals within paratuberculosis-infected herds.

## 1. Introduction

*Mycobacterium avium* subspecies (subsp.) *paratuberculosis* (MAP), a slow growing, obligate intracellular pathogen, is the etiologic agent of paratuberculosis, also known as Johne's disease (JD) (1). It is a chronic granulomatous infection causing relevant economic losses in the cattle industry because of reduced milk production, weight loss, and eventual death (2–4). MAP is commonly transmitted by the fecal-oral route (2, 4, 5). The high resistance of MAP, the long incubation period, and the pathogenesis of the disease make the management of the disease difficult (6). In addition, the difficulty, in the absence of accurate diagnostic tests entail delayed diagnosis of JD, of making an early diagnosis does not allow prevention of MAP diffusion within cattle herds, nor protection of young calves that are more susceptible to infection (2). After the long incubation period, the animals can be divided into: (i) infected, when MAP is present intracellularly in animal tissues, (ii) infectious, when the animal is shedding MAP, and (iii) affected, when animals show clinical signs (7), which are visible after 2–6 years post-infection. Different tests, such as fecal polymerase chain reaction (PCR) and enzyme-linked immunosorbent assay (ELISA) are commonly used to make JD diagnosis (8). Unfortunately, cattle become positive to these tests only during the late sub-clinical phase, and thus, the successful early diagnosis of JD remains a challenge, especially if considering the low efficacy of laboratory tests (7). Over the last years, proteomics and metabolomics of animal sera provided encouraging results (9–11) and different studies on infected cattle have been carried out to introduce new tools with the aim of unraveling important molecular markers that describe the different stages of JD, diagnose early MAP infection, and overcome the lack of reliable tests (12–14). Spectrometric and spectroscopic methods recently were used to investigate the metabolic changes in naturally and experimentally MAP-infected cattle (13, 15–17). In details, the metabolomics studies, carried out on sera of experimentally MAP-infected cattle using ^1^H nuclear magnetic resonance (NMR) spectrometry, showed metabolomics changes related to energy shortages, increased fat metabolism, and altered protein turnover (13). Taylor et al. first examined the sera metabolic profiles of naturally MAP-infected Holstein-Friesian heifers and compared them to controls, finding changes in some amino acids related to biochemical reprogramming (16) and then proposed polyunsaturated fatty acids and eicosanoids as potential biomarkers for MAP diagnosis (18). In our previous study, we applied direct analysis in real time high resolution mass spectrometry (DART-HRMS) coupled to a mid-level data fusion approach to tease out molecular features able to chemically characterize the animal sera at the infected and infectious stage (15).

The least absolute shrinkage and selection operator (LASSO) method yields multiclass classifiers that involve only a small subset of discriminant metabolites (19–21). A mathematical weight for each statistically informative metabolite is calculated by LASSO, based on its capability in characterizing a certain class. The application of LASSO method already showed its utility in multiclass classification of cancer tissues for rapid diagnosis in intra-surgery settings upon integration with mass spectrometry (20, 22, 23). Recently, LASSO showed good performances in classifying biological samples based on the molecular information captured by NMR (24–26). Note that this parsimonious method is less susceptible to the noise linked to the heterogeneity of the samples (27). In the current study, the combination of blood chemistry and ^1^H NMR signature analysis, upon integration with LASSO method, was adopted to robustly determine active signatures of metabolic changes capable of classifying infected (non-infectious), infectious, and negative (control) cattle. Finally, pathway analysis was performed on the informative metabolites teased out by LASSO to reveal potential dysregulated metabolisms.

## 2. Methods

### 2.1. Animal selection

Holstein Friesian cattle were selected from four dairy farms of the Veneto region (Italy) with known paratuberculosis initial seroprevalences of >10% and were divided into age-cohorts by reproduction cycle: heifers, primiparous, and pluriparous cows. A total of 356 animals were monitored for up to 4 lactations. Blood and fecal sample were collected at 30 ± 15 days before the expected calving date to minimize individual metabolic variations, except for young heifers that were recruited at 13–15 months of age. During the mid-dry period, cows do not produce milk and the metabolic-hormonal changes that lead to calf delivery are not fully established. MAP-affected animals and cattle showing other concurrent diseases or under pharmacological treatment were excluded from the study. Blood sample collection was performed under authorization n. 506/2015 of the Italian Ministry of Health for the use of animals for experimental purposes.

### 2.2. Sample collection and testing for JD

Blood samples were collected from the jugular vein in anticoagulant-free vacutainer tubes [Greiner Bio-One (Kremsmünster, Austria)], left to coagulate at room temperature for 2–4 h, and centrifuged at 3,000 x g for 5 min. Aliquots from the sera obtained were used for detecting serum antibodies against MAP using a commercial ELISA (IDEXX Paratuberculosis Screening Ab, IDEXX Laboratories, Inc. Westbrook, MN, USA) and applying the manufacturer's instructions for analysis. Inconclusive and positive sera were submitted to an ELISA biphasic confirmation test (IDEXX Paratuberculosis Verification Ab, IDEXX Laboratories, Inc. Westbrook, MN, USA).

Individual fecal samples were collected from the rectal ampulla and analyzed applying microbiological and molecular diagnostic methods for MAP identification. All samples were processed for testing by IS900 direct real-time PCR (qPCR, Applied Biosystems, Nieuwerkerk a/d IJssel, The Netherlands) according to Pozzato et al. (28, 29) while the culture was carried out by a double decontamination method on modified Middlebrook 7H9 liquid media (7H9+). After 6 weeks of culture, 7H9+ broths were examined by Ziehl-Nielsen staining and real-time PCR, as reported by our previous work (27a). One fecal aliquot from each animal was stored at −80°C for possible future analyses.

### 2.3. JD health status assignment and sample selection for ^1^H NMR analysis and for blood indices determination

From 356 animals, a total of 854 serum samples were collected during the study period, resulting in a mean value of 2.40 samples per cattle (range 1–5). Regarding JD testing, the frequency of positive animals throughout the study period was 6.23% by serology and 11.05% by fecal PCR/culture. At the end of the prospective study, the status of infectious was assigned to serum samples of those animals that tested positive fecal PCR or culture. To exclude “passive shedders” (30) and assure higher confidence of the status we decided to select those animals that had seroconverted for MAP by ELISA as well. Assuming that the infection occurred in the 1st year of life, the status of infected (non-infectious) was retrospectively assigned to the previous sample of the bovines classified as infectious, in which all JD tests (ELISA, PCR, and culture) produced negative results. The status of negative was eventually assigned to exposed cohort animals from the same infected herds that repeatedly tested negative along the study period and exhibited at least one subsequent JD negative result after the selected sampling date. These control animals were matched to cases according to sampling date and age category (heifers, primiparous cows, and pluriparous cows) in order to minimize the variability due to dietary and management conditions. The average number of samplings for these animals was 2.84 (range 2–4). From the collection of sera stored at −80°C, 23 sera of infectious animals, 10 sera of infected animals and 26 sera of negative animals were selected and then submitted to ^1^H NMR analysis and blood indices determination. The age of the selected animals averaged 53.5 months (ranging between 13 and 119 months).

### 2.4. ^1^H NMR sample preparation and analysis

Dried serum sample was reconstituted with 200 μL of ultra pure water. Subsequently, 50 μL of a standard buffer solution (54% D_2_O: 46% 810 mM KH_2_PO_4_ pH 7.0 v/v containing 5 mM DSS (2,2-dimethyl-2-silcepentane-5-sulphonate), 5.84 mM 2-chloropyrimidine-5 carboxylate, and 0.1% NaN_3_ in H_2_O) was added to the reconstituted sample. The diluted, buffered sample (250 μL) was then transferred to 3 mm SampleJet NMR tube for subsequent spectral analysis. All ^1^H-NMR spectra were collected on a 700 MHz Avance III (Bruker Daltonics, Bremen, Germany) spectrometer equipped with a 5 mm HCN Z-gradient pulsed-field gradient (PFG) cryoprobe. The ^1^H-NMR spectra were acquired at 25°C using the first transient of the NOESY pre-saturation pulse sequence (noesy1dpr), chosen for its high degree of quantitative accuracy. All free induction decays (FIDs) were zero-filled to 250 K data points. The singlet produced by the DSS methyl groups was used as an internal standard for chemical shift referencing (set to 0 ppm) and for quantification.

### 2.5. NMR quantification using MagMET

For quantification, all ^1^H-NMR spectra were processed and analyzed using a MagMET software package developed in-house (31). MagMET allows for qualitative and quantitative analysis of an NMR spectrum by automatically fitting spectral signatures from an internal database to the spectrum. Specifically, the spectral fitting for metabolites was done using the standard serum metabolite library. Typically, all of the visible peaks were assigned. Most of the visible peaks were annotated with a compound name. It has been previously shown that this fitting procedure provides absolute concentration accuracy of 90% or better (31).

### 2.6. Blood indices

Biochemical indices were determined in serum samples using commercial dedicated kits applied to the automated clinical chemistry analyzer, Cobas C501 (Roche Diagnostics, Mannheim, Germany). Non-esterified fatty acid (NEFA) and β-hydroxybutyrate (BHB) were determined with a colorimetric kit produced by Randox (Randox Laboratories Ltd, Crumlin, UK), whereas haptoglobin (Hp) concentration was obtained by using the reagents from the Tridelta Phase Haptoglobin Colorimetric Assay (Tridelta Development Limited, Maynooth, County Kildare, Ireland.): NEFA, BHB and Hp were analyzed following the manufacturer's specific application for the Cobas C501 analyzer. Almost 200 μL of each sample were necessary for biochemical analysis as dead volume, but only 2.0, 6.0, and 3.8 μL were used to measure NEFA, b-OHB and HP, respectively. Serum electrophoresis was performed on a semi-automated agarose gel system (Hydrasys LC Sebia, Bagno a Ripoli, FI, Italy). Serum electrophoresis was performed to evaluate A/G ratio and acute phase protein pattern. Only 10 μL were used for serum protein electrophoresis while 2.0 μL were used for total protein analysis on Cobas 501. The percentage of the each protein fraction, determined by electrophoretic analysis, was converted into the absolute concentration (g/L) based on the total protein concentration obtained by the biuret method on the Cobas C501 analyzer.

### 2.7. Statistical analysis

**Figure 1:**
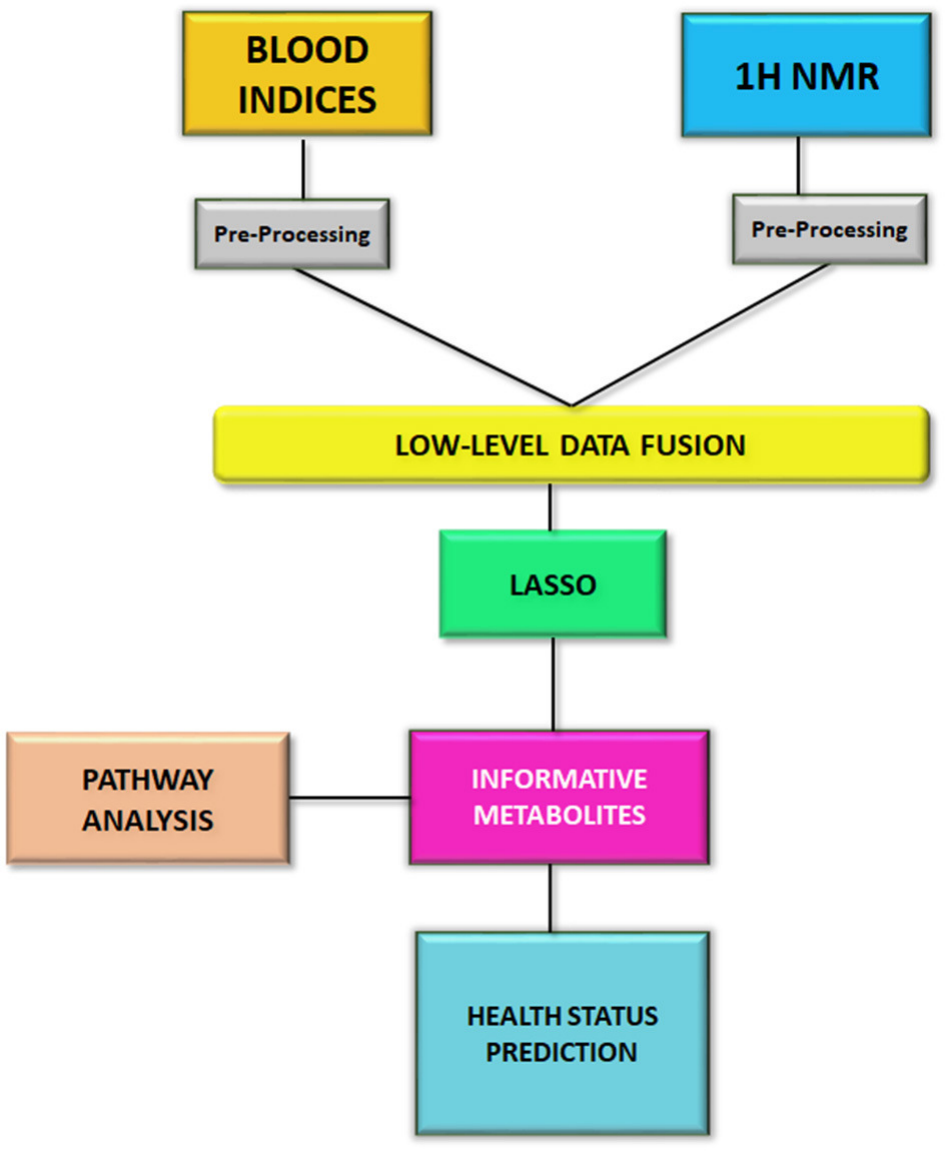
The workflow of this study.

The statistical analysis was performed by using RStudio software 4.0.2 with caret package (32), a useful package for model implementation. The processed ^1^H NMR data were autoscaled, while the blood chemistry data were imputed by k-nearest neighbor and then autoscaled. Afterwards, the two datasets were concatenated by low-level data fusion. Low-level data fusion is a simple concatenation of data in a unique table (33–36). The merged dataset was submitted to the LASSO method, a multinomial regression with L1 penalty optimized by a grid search, with the aims of i) selecting and shrinking variables, and ii) predicting the health status of animals, i.e., negative, MAP-infected, or MAP-infectious. Moreover, in order to select the best classifier generated by LASSO, a 10-time repeated 5-fold cross validation was performed on the concatenated data using the retrieved informative features. To this aim, we split the data in 75% of data for training the model (47 animals) and 25% for testing it (12 animals). For each iteration, the training/test split was different. In the first iteration, the model was tested on test data (12 animals) and test errors were calculated. After 50 iterations, the average of the test errors was determined and sensitivity (true positive rate), specificity (true negative rate) and accuracy were calculated. The statistical workflow of this study is reported in Figure 1.

### 2.8. Pathway analysis

Metabolic pathway analysis was performed using the “pathway analysis” section of the web platform at www.metaboanalyst.ca. After loading the discriminating metabolites teased out by the LASSO, and the metabolites' quantitative values (concentrations obtained by NMR and blood chemistry), the metabolic pathway analysis was performed in order to investigate the potential metabolic pathways that could significantly have relevance in infected and infectious animals. The pathway analysis is a form of topology analysis, and applies a global test algorithm (37) for differentially testing metabolites in functionally related groups and relative-betweeness-centrality to estimate the importance of a compound in a given metabolic pathway. Since the pathway analysis on the Metaboanalyst web platform allows only a binary comparison, we compared (i) MAP-infected cattle vs. negative animals and ii) MAP-infectious cattle vs. negative animals. According to both the resulting *p*-value and the impact value, the analysis graphically shows the pathways that could be potentially altered between each pair of compared MAP stages. The pathway analysis provides for each pathway: (i) a list of the matched metabolites over the total number of metabolites, (ii) the raw *p*-value, (iii) the *p*-value adjusted by Holm–Bonferroni method, (iv) the *p*-value adjusted by the false discovery rate (FDR), and v) the pathway impact value. In the plot, the top pathways are ranked by adjusted *p*-values (y-axis) and the total number of hits that determine the impact of the pathway (x-axis). Cut-off values for pathway analyses results were: impact score >0.1, false discovery rate (FDR) < 0.25 and *p*-value < 0.05. The threshold of FDR < 0.25 denotes the confidence of ‘possible’, while the threshold of FDR < 0.05 is regarded as ‘high confidence’ (38).

Moreover, the node color is based on the relevant *p*-value, and the node radius is determined based on its pathway impact value. Large radius means high impact value, small radius means low impact value. Finally, the color graduates from white (high *p*-value) to yellow, orange, and red (low *p*-value).

## 3. Results

### 3.1. Significant metabolites

**Figure 2:**
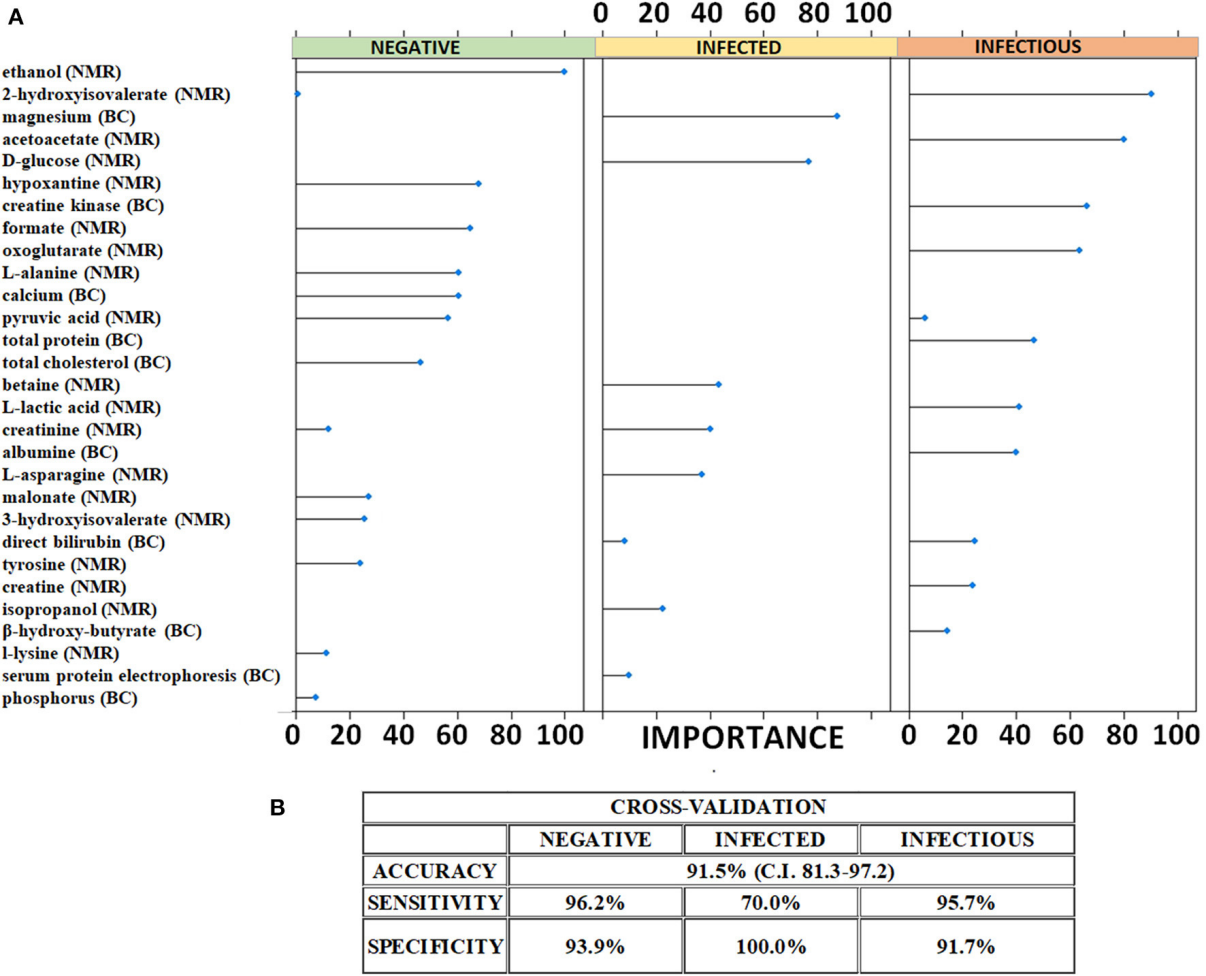
Predictive metabolic features and performances of the LASSO classifier. **(A)** Metabolites selected as indicative of negative, MAP-infected and MAP-infectious animals by LASSO. The related weights of each significant metabolites are illustrated. **(B)** Performances of the LASSO classifier on cross-validation. The values of the statistical indicators were calculated within the 95% confidence interval.

The list of metabolites quantified in the analyzed samples by ^1^H-NMR and blood chemistry test is reported in Supplementary Table S1. After low-level data fusion, LASSO method retrieved and validated the statistically significant metabolites (Figure 2A) within a cross-validation approach. The LASSO has the capability of minimizing the statistically significant variables and, thus, tease out a small pattern of metabolites capable of discriminating each study group. The LASSO was able to effectively shrink the amount of diagnostically significant metabolites by selecting a total of 29 molecules that could reliably predict the health status (with respect to MAP infection) of the animals. Figure 2A and Supplementary Table S2 reports the list of metabolites and the weights assigned by the LASSO. A mathematical weight for each statistically informative feature is calculated by the LASSO depending on the importance of the concentration (obtained by NMR and blood chemistry) to a class. Features that did not contribute to discriminating a class received a weight of zero and were discarded. Each metabolite whose concentration was important for characterizing the three specific classes was given a high weight. As shown in Figure 2A, magnesium, D-glucose, betaine, creatinine, L-asparagine, and isopropanol were the variables that allowed the LASSO to predict that a sample belonged to infected animals. In the same vein, the metabolic pattern of hydroxyisovalerate, acetoacetate, creatine kinase, oxoglutarate, total protein, lactic acid, albumin, bilirubin, creatine, and β-hydroxybutyrate was indicative of infectious cattle.

### 3.2. LASSO classifier

These selected molecular features were used to build up a classifier that was cross-validated. The results of the cross-validation are reported in Figure 2B. A repeated k-fold cross-validation was performed for the model evaluation. The results of the cross-validation are shown in the confusion matrix reported in the Supplementary Table S3. The classifier correctly classified 25/26 control sera from negative, 7/10 sera from infected and 22/23 sera from infectious animals. Based on the cross-validation results, we calculated the statistical indicators. The LASSO classifier achieved an overall accuracy of 91.5% (95% CI: 81.3–97.2) with high values of sensitivity and specificity for each class. Specifically, it showed sensitivity of 96.2% and specificity of 93.9% in correctly predicting negative animals. Infected animals were correctly predicted by the LASSO classifier with a very high specificity (100%) and a moderate sensitivity (70%). Finally, the classifier also categorized infectious cattle with a sensitivity of 96% and a specificity of 92%.

### 3.3. Pathway analysis

**Figure 3:**
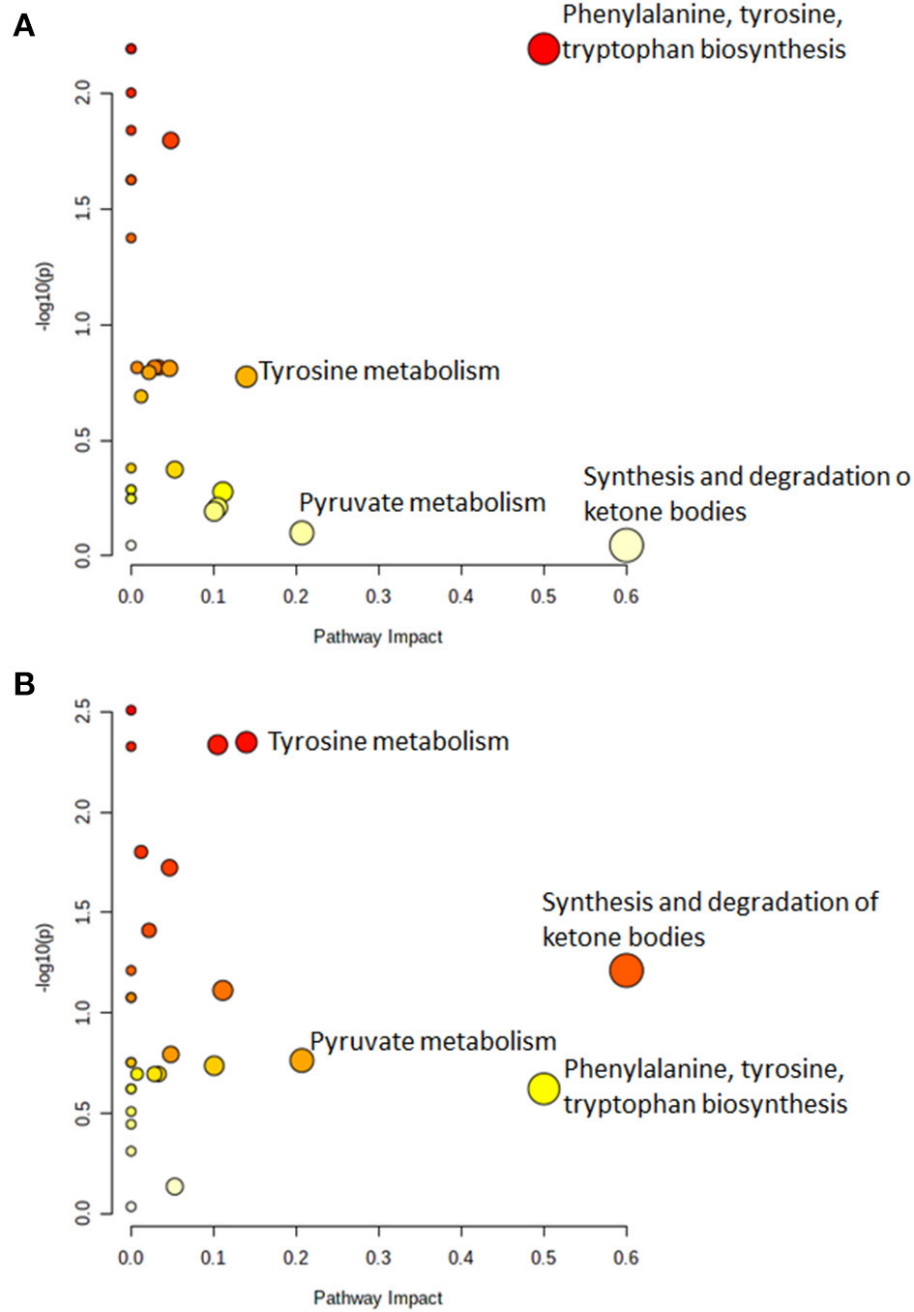
The metabolic pathway analysis identified differences between **(A)** MAP-infected vs. negative cattle and **(B)** MAP-infectious vs. negative cattle. In each plot, the top pathways were ranked by adjusted *p*-values (y-axis) and the total number of hits that determined the impact of the pathway (x-axis). Moreover, the node color was based on its *p*-value and the node radius was determined based on the pathway impact values. Large radius means high impact value, small radius means low impact value. Finally, the color graduates from white (high *p*-value) to yellow, orange, and red (low *p*-value). Cut-off values for pathway impact score >0.1, false discovery rate (FDR) < 0.25 and *p*-value < 0.05.

Figure 3 shows the significantly altered pathways in both infected and infectious cattle. Specifically, the dysregulated pathways (considering cut-off values for pathway impact score >0.1, false discovery rate (FDR) < 0.25 and *p-*value < 0.05) in infected cattle, is the phenylalanine, tyrosine, and tryptophan biosynthesis (Figure 3A). In infectious cattle, the synthesis and degradation of ketone bodies and tyrosine metabolism were the most relevant metabolome pathways potentially involved in the observed variation of serum metabolites (Figure 3B). All the *p-*values, FDR and impact values are reported in Supplementary Tables S4, S5 of the Supplementary material.

## 4. Discussion

Early diagnosis of JD is a considerable challenge (7). This is the reason why several authors have recently applied some combination of new methods (13, 15–17) to provide early evidence of MAP infection in cattle, for which common diagnostic tests fail. In this study, we verified the capability of ^1^H NMR combined with blood chemistry to effectively distinguish between negative, naturally infected, and infectious animals, and to identify some metabolites that could be associated with the latent stage of infection by MAP. The discovery of unexpectedly dysregulated pathways are notably more successful when non-targeted analyses are performed, and such discovery is enhanced by the combination of data sources (39). In the present work, statistical analysis using a LASSO allowed establishing possible molecular markers of disease and categorizing serum samples into the animals' JD status with high accuracy. In this study, we successfully captured the characteristic metabolic profiles of healthy, infected, and infectious animals, and thus, identified potentially altered metabolic pathways associated with these three different health statuses. Our pathway analysis revealed MAP-infected cattle have perturbed phenylalanine, tyrosine and tryptophan biosynthesis: these observations are in accordance with De Buck et al., who reported significant increases in the amino acid tyrosine (together with threonine, isoleucine, leucine, and asparagine) in experimentally MAP-infected cattle (13). On the other hand, Tata et al. found increased tryptamine levels in MAP-infected and infectious cattle, as compared to controls (15). In the same vein, we observed an altered metabolism of the amino acid tyrosine in infectious animals (Figure 3B). The alterations in amino acid metabolism could be due to their utilization by immune-cells or restrictive absorption by digestive system (17). The altered synthesis and degradation of ketone bodies revealed by our pathway analysis was already reported in 2014 by De Buck et al. in experimentally infected cattle (13). This is consistent with an energy deficit and the greater mobilization of lipid stores. Note that the synthesis and degradation of the ketone body metabolic pathway is known to be up-regulated when glucose sources are severely restricted, and an excess of ketone bodies are consequently produced. While the ketone body, acetone, was one of the most discriminatory metabolites in MAP-infected cattle in the previous study carried out in 2014 (13), we did not observe significant changes in acetone concentration in our current raw data, nor after we had applied the LASSO method to these data. Note that acetone is a very volatile molecule that can be easily lost from samples. As reported in Figure 3B, we also observed increased pyruvate metabolism in infectious cattle, which we observed also in MAP-infected cattle, and with a minor impact and *p-*value. This is likely due to the alteration of glycolytic metabolism in these cattle.

Some limitations set our metabolic signature, with ^1^H NMR and blood indices, apart from metabolic biomarker discovery that can be used in routine analysis: (i) the small sample size of clinical specimens in the statistical validation step to assess the prediction ability of the method; (ii) the potential influence of diet and herd management on the metabolic fingerprint. While we are confident that the retrospective assignment of the infectious animals is highly reliable, we cannot exclude that the animals categorized as negative could have lately turned to a different status.

## 5. Conclusion

The proposed approach allowed to move one step forward the understanding and diagnosis of MAP. Alterations of amino acid metabolism were observed. While the tyrosine metabolism was significantly perturbed in infectious bovines, the biosynthesis of phenylalanine, tyrosine, and tryptophan was dysregulated in those infected. In accordance with previous scientific findings in experimentally infected cattle a perturbation of the synthesis and degradation of ketone bodies in infectious animals was also confirmed. Further investigations are being carried out to validate the method on new samples and thus evaluate the effect of diet and herd management on the informative markers.

## Data availability statement

The raw data supporting the conclusions of this article will be made available by the authors, without undue reservation.

## Ethics statement

The animal study was reviewed and approved by Italian Ministry of Health with the authorization no. 506/2015. Written informed consent was obtained from the owners for the participation of their animals in this study.

## Author contributions

RP, JD, and NP: conceived, designed, and supervised the research. AT and IP: analyzed the data and wrote the main manuscript text and made the Figures 1–3. MB, LP, BD, AS, and VB: performed the research. AM: statistically analyzed the data and provided new statistical tools. All authors read and approved the manuscript.
