# Supplementary material for: Metabolic signature of Mycobacterium avium subsp. paratuberculosis infected and infectious dairy cattle by integrating nuclear magnetic resonance analysis and blood indices
Source: Front Vet Sci. 2023 Apr 17;10:1146626. doi: 10.3389/fvets.2023.1146626 (PMC10150450; doi:10.3389/fvets.2023.1146626)
Supplement: Supplementary file 1 [file Table_1.DOCX]

Supplementary Material

**Prediction of latent paratubercolosis infection in dairy cattle by integrating nuclear magnetic resonance analysis and blood indices.**

**Andrea Massaro^1$^ , Alessandra Tata^1$^ , Ivana Pallante^2^ , Valentina Bertazzo^3^ , Massimo Bottazzari^4^ , Laura Paganini^4^ , Brunella Dall’Ava^4^ , Annalisa Stefani^3^ , Jeroen De Buck^5^ , Roberto Piro^1^ , Nicola Pozzato^2^.**

^1^ Istituto Zooprofilattico Sperimentale delle Venezie, Laboratorio di Chimica Sperimentale, Vicenza, Italy

^2^ Istituto Zooprofilattico Sperimentale delle Venezie, Laboratorio di Medicina Forense Veterinaria, Vicenza, Italy

^3^ Istituto Zooprofilattico Sperimentale delle Venezie, Medicina di Laboratorio, Legnaro, Italy

^4^ Istituto Zooprofilattico Sperimentale delle Venezie, Laboratorio di Diagnostica Clinica e Sierologia di Piano, Verona, Italy

^5^ Faculty of Veterinary Medicine, University of Calgary, Alberta, Canada

*** Correspondence:**Alessandra Tata [atata@izsvenezie.it](mailto:atata@izsvenezie.it)

**Supplementary table 1. Lists of all the metabolites detected by NMR and blood chemistry.**

| \| **List of metabolites quantified by NMR** \| \|  \| **List of metabolites quantified by blood chemistry** \| \| \| \| \| \| --- \| --- \| --- \| --- \| --- \| --- \| --- \| --- \| \| 1-Methylhistidine \|  \|  \| Non-esterified fatty acids \| \|  \|  \|  \| \| 2-Hydroxybutyrate \|  \|  \| Albumin \|  \|  \|  \|  \| \| Acetic acid \|  \|  \| Aspartate amino-transferase \| \| \|  \|  \| \| Betaine \|  \|  \| Beta hydroxybutyrate \| \|  \|  \|  \| \| Acetoacetate \|  \|  \| Direct bilirubin \| \|  \|  \|  \| \| L-Carnitine \|  \|  \| Total bilirubin \| \|  \|  \|  \| \| Creatine \|  \|  \| Calcium \|  \|  \|  \|  \| \| Dimethylglycine \|  \|  \| Total cholesterol \| \|  \|  \|  \| \| Citric acid \|  \|  \| Creatine kinase \| \|  \|  \|  \| \| Choline \|  \|  \| Serum protein electrophoresis \| \| \|  \|  \| \| Ethanol \|  \|  \| Phosphorus \|  \|  \|  \|  \| \| D-Glucose \|  \|  \| Gamma glutamyl transferase - ggt \| \| \| \|  \| \| Glycine \|  \|  \| Globuline \|  \|  \|  \|  \| \| Glycerol \|  \|  \| Glucose \|  \|  \|  \|  \| \| Formate \|  \|  \| Magnesium \|  \|  \|  \|  \| \| L-Glutamic acid \|  \|  \| Total protein \| \|  \|  \|  \| \| Hypoxanthine \|  \|  \| Urea \|  \|  \|  \|  \| \| Tyrosine \|  \|  \|  \|  \|  \|  \|  \| \| L-Phenylalanine \|  \|  \|  \|  \|  \|  \|  \| \| L-Alanine \|  \|  \|  \|  \|  \|  \|  \| \| L-Proline \|  \|  \|  \|  \|  \|  \|  \| \| L-Threonine \|  \|  \|  \|  \|  \|  \|  \| \| L-Asparagine \|  \|  \|  \|  \|  \|  \|  \| \| D-Mannose \|  \|  \|  \|  \|  \|  \|  \| \| Isoleucine \|  \|  \|  \|  \|  \|  \|  \| \| L-Histidine \|  \|  \|  \|  \|  \|  \|  \| \| L-Lysine \|  \|  \|  \|  \|  \|  \|  \| \| L-Serine \|  \|  \|  \|  \|  \|  \|  \| \| L-Lactic acid \|  \|  \|  \|  \|  \|  \|  \| \| L-Acetylcarnitine \|  \|  \|  \|  \|  \|  \|  \| \| Oxoglutarate \|  \|  \|  \|  \|  \|  \|  \| \| L-Ornithine \|  \|  \|  \|  \|  \|  \|  \| \| Pyruvic acid \|  \|  \|  \|  \|  \|  \|  \| \| Succinate \|  \|  \|  \|  \|  \|  \|  \| \| Sarcosine \|  \|  \|  \|  \|  \|  \|  \| \| 3-Hydroxybutyric acid \|  \|  \|  \|  \|  \|  \|  \| \| 2-hydroxyisovalerate \|  \|  \|  \|  \|  \|  \|  \| \| L-Alpha-aminobutyric acid \| \|  \|  \|  \|  \|  \|  \| \| 3-Methyl-2-oxovaleric acid \| \|  \|  \|  \|  \|  \|  \| \| L-arginine \|  \|  \|  \|  \|  \|  \|  \| \| Creatinine \|  \|  \|  \|  \|  \|  \|  \| \| L-Glutamine \|  \|  \|  \|  \|  \|  \|  \| \| L-Leucine \|  \|  \|  \|  \|  \|  \|  \| \| Malonate \|  \|  \|  \|  \|  \|  \|  \| \| Ketoleucine \|  \|  \|  \|  \|  \|  \|  \| \| Methionine \|  \|  \|  \|  \|  \|  \|  \| \| 3-Hydroxyisovaleric acid \|  \|  \|  \|  \|  \|  \|  \| \| Isopropanol \|  \|  \|  \|  \|  \|  \|  \| \| Valine \|  \|  \|  \|  \|  \|  \|  \| \| Acetone \|  \|  \|  \|  \|  \|  \|  \| \| Isobutyric acid \|  \|  \|  \|  \|  \|  \|  \| \| Methanol \|  \|  \|  \|  \|  \|  \|  \| \| Propylene glycol \|  \|  \|  \|  \|  \|  \|  \| \| Dimethyl sulfone \|  \|  \|  \|  \|  \|  \|  \| |
| --- | --- | --- | --- | --- | --- | --- | --- | --- | --- | --- | --- | --- | --- | --- | --- | --- | --- | --- | --- | --- | --- | --- | --- | --- | --- | --- | --- | --- | --- | --- | --- | --- | --- | --- | --- | --- | --- | --- | --- | --- | --- | --- | --- | --- | --- | --- | --- | --- | --- | --- | --- | --- | --- | --- | --- | --- | --- | --- | --- | --- | --- | --- | --- | --- | --- | --- | --- | --- | --- | --- | --- | --- | --- | --- | --- | --- | --- | --- | --- | --- | --- | --- | --- | --- | --- | --- | --- | --- | --- | --- | --- | --- | --- | --- | --- | --- | --- | --- | --- | --- | --- | --- | --- | --- | --- | --- | --- | --- | --- | --- | --- | --- | --- | --- | --- | --- | --- | --- | --- | --- | --- | --- | --- | --- | --- | --- | --- | --- | --- | --- | --- | --- | --- | --- | --- | --- | --- | --- | --- | --- | --- | --- | --- | --- | --- | --- | --- | --- | --- | --- | --- | --- | --- | --- | --- | --- | --- | --- | --- | --- | --- | --- | --- | --- | --- | --- | --- | --- | --- | --- | --- | --- | --- | --- | --- | --- | --- | --- | --- | --- | --- | --- | --- | --- | --- | --- | --- | --- | --- | --- | --- | --- | --- | --- | --- | --- | --- | --- | --- | --- | --- | --- | --- | --- | --- | --- | --- | --- | --- | --- | --- | --- | --- | --- | --- | --- | --- | --- | --- | --- | --- | --- | --- | --- | --- | --- | --- | --- | --- | --- | --- | --- | --- | --- | --- | --- | --- | --- | --- | --- | --- | --- | --- | --- | --- | --- | --- | --- | --- | --- | --- | --- | --- | --- | --- | --- | --- | --- | --- | --- | --- | --- | --- | --- | --- | --- | --- | --- | --- | --- | --- | --- | --- | --- | --- | --- | --- | --- | --- | --- | --- | --- | --- | --- | --- | --- | --- | --- | --- | --- | --- | --- | --- | --- | --- | --- | --- | --- | --- | --- | --- | --- | --- | --- | --- | --- | --- | --- | --- | --- | --- | --- | --- | --- | --- | --- | --- | --- | --- | --- | --- | --- | --- | --- | --- | --- | --- | --- | --- | --- | --- | --- | --- | --- | --- | --- | --- | --- | --- | --- | --- | --- | --- | --- | --- | --- | --- | --- | --- | --- | --- | --- | --- | --- | --- | --- | --- | --- | --- | --- | --- | --- | --- | --- | --- | --- | --- | --- | --- | --- | --- | --- | --- | --- | --- | --- | --- | --- | --- | --- | --- | --- | --- | --- | --- | --- | --- | --- | --- | --- | --- | --- | --- | --- | --- | --- | --- | --- | --- | --- | --- | --- | --- | --- | --- | --- | --- | --- | --- | --- | --- | --- | --- | --- | --- | --- | --- | --- | --- | --- | --- | --- | --- | --- | --- | --- | --- | --- | --- | --- | --- | --- | --- | --- | --- | --- | --- | --- | --- | --- |

**Supplementary Table 2. List of LASSO weights of significant features**

| **Features** | **Negative** | **Infected** | **Infectious** |
| --- | --- | --- | --- |
| **ethanol (NMR)** | 100 | 0 | 0 |
| **hypoxantine (NMR)** | 67,7658156 | 0 | 0 |
| **formate (NMR)** | 64,7273531 | 0 | 0 |
| **L-alanine (NMR)** | 60,3996351 | 0 | 0 |
| **calcium (BC)** | 60,2932866 | 0 | 0 |
| **pyruvic acid (NMR)** | 56,6141553 | 0 | 6,08693727 |
| **total cholesterol (BC)** | 46,1622532 | 0 | 0 |
| **malonate (NMR)** | 27,1283612 | 0 | 0 |
| **3-hydroxyisovalerate (NMR)** | 25,3421795 | 0 | 0 |
| **tyrosine (NMR)** | 23,9760153 | 0 | 0 |
| **creatine (NMR)** | 11,9692535 | 40,0370898 | 0 |
| **l-lysine (NMR)** | 11,0658416 | 0 | 0 |
| **phosphorus (BC)** | 7,42991687 | 0 | 0 |
| **2-hydroxyisovalerate (NMR)** | 0,50934476 | 0 | 90,3419395 |
| **betaine (NMR)** | 0 | 43,2113475 | 0 |
| **acetoacetate (NMR)** | 0 | 0 | 80,1030398 |
| **creatinine (NMR)** | 0 | 0 | 23,6188212 |
| **glucose (NMR)** | 0 | 76,813373 | 0 |
| **L-asparagine (NMR)** | 0 | 36,8949118 | 0 |
| **L-lactic acid (NMR)** | 0 | 0 | 40,9705568 |
| **oxoglutarate (NMR)** | 0 | 0 | 63,3916222 |
| **isopropanol (NMR)** | 0 | 22,5478965 | 0 |
| **albumine (BC)** | 0 | 0 | 39,9135661 |
| **β-hydroxy-butyrate (BC)** | 0 | 0 | 14,2128608 |
| **direct bilirubin (BC)** | 0 | 8,17862795 | 24,5272899 |
| **creatine kinase (BC)** | 0 | 0 | 66,305592 |
| **serum protein electrophoresis (BC)** | 0 | 9,75780164 | 0 |
| **magnesium (BC)** | 0 | 87,224833 | 0 |
| **total protein (BC)** | 0 | 0 | 46,7599017 |

**Supplementary Table 3. LASSO Confusion matrix**

|  |  | ***Actual class*** | | |
| --- | --- | --- | --- | --- |
|  |  | **NEGATIVE** | **INFECTED** | **INFECTIOUS** |
| ***Predicted class*** | **NEGATIVE** | **25** | **1** | **1** |
|  | **INFECTED** | **0** | **7** | **0** |
|  | **INFECTIOUS** | **1** | **2** | **22** |

**Supplementary Table 4. Pathways list of MAP infected cows**

**Supplementary Table 5. Pathways list of MAP infectious cows**
